# The role of hydrogen peroxide in hip arthroplasty: A narrative review

**DOI:** 10.1177/1750458921996259

**Published:** 2021-07-11

**Authors:** Andrew Kailin Zhou, Milind Girish, Azeem Thahir, Jiang An Lim, Caitlyn Tran, Shaan Patel, Matija Krkovic

**Affiliations:** 1 Department of Trauma and Orthopaedics, Addenbrookes Major Trauma Unit, Cambridge University Hospitals, Cambridge, UK; 2 School of Clinical Medicine, University of Cambridge, Cambridge, UK; 3 Department of Medicine, Institute for Society and Genetics, University of California, Los Angeles, USA

**Keywords:** Arthroplasty, Hydrogen peroxide, Prosthesis loosening, Irrigation, Embolism

## Abstract

Hydrogen peroxide has become more commonly used in hip arthroplasties due to high risk of periprosthetic infections. Its purported roles include irrigation, haemostasis, reduction of aseptic loosening and attachment of antibiotics. However, current literature does not provide conclusive evidence on the efficacy of hydrogen peroxide in preventing aseptic loosening, with some controversy around whether it in fact contributes to aseptic loosening. The complications of hydrogen peroxide across medicine are well distinguished; however, the risks within orthopaedic surgery and hip arthroplasties are not well known. Beyond cytotoxicity, the most dangerous reported risk associated with hydrogen peroxide in hip arthroplasties was an oxygen embolism in an unvented femoral canal and acrylic bone cement, consequentially leading to cardiac arrest. However, it may be inappropriate to solely attribute the oxygen embolism to the use of hydrogen peroxide and thus if used appropriately, hydrogen peroxide may have a justifiable role in hip arthroplasty surgery. In this narrative review, we present the current uses of hydrogen peroxide while evaluating its associated risks. We have summarised the key indications and aggregated recommendations to provide guidelines for the use of hydrogen peroxide in hip arthroplasty.

**Provenance and Peer review:** Unsolicited contribution; Peer reviewed; Accepted for publication 30 January 2021.

## Introduction

Due to the notably high risk, 1% of hip arthroplasty cases experience periprosthetic infection (Akgün et al 2019). Of periprosthetic infection in hip arthroplasties, hydrogen peroxide is now commonly used in hip arthroplasties, and its role varies from being used in irrigation, haemostasis and antisepsis to reduction of aseptic loosening and attachment of antibiotics. (Ackland et al 2009, Ketonis et al 2009, Yang et al 2016). Hydrogen peroxide’s antiseptic mechanisms are thought to involve deoxyribose nucleic acid (DNA) damage as well as lipid and protein peroxidation, although in vitro illustrations these may differ significantly from the in vivo environment where antimicrobial action may be diluted by regional bodily fluids or the action of catalase that is present in normal human tissue. Beyond their explicit antimicrobial mechanisms, evidence also suggests they can reduce bacterial biofilm production (DeQueiroz & Day 2007, Glynn et al 2009). Biofilms form a hugely significant concern within orthopaedic surgery; particularly at the surface of implants where they can protect microflora from antibiotics and host immune mechanisms. Indeed, biofilms have been labelled as one of the most critical steps to the pathogenesis of periprosthetic joint infections (Flemming et al 2016).

**Figure 1:**
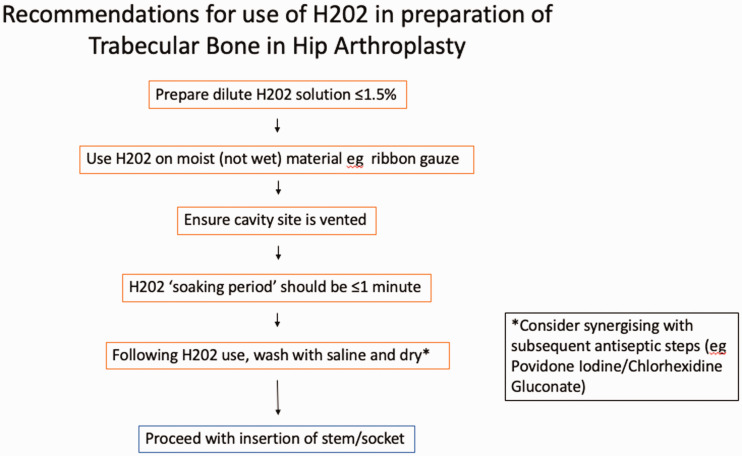
Recommendations for use of H202 in preparation of trabecular bone in hip arthroplasty.

However, current literature does not provide conclusive evidence on many of these benefits; for example, the efficacy of hydrogen peroxide in aseptic loosening, defined as the failure of bonding between the hip implant and acetabulum in the absence of infection (Apostu et al 2018). There has been much controversy around this aspect, and some studies suggest that hydrogen peroxide in fact contributes to aseptic loosening (Guerin et al 2006). A thorough literature search was performed in PubMed, Medline, Embase and Cochrane databases using the keywords hydrogen peroxide, hip and arthroplasty. All abstracts of retrieved articles were reviewed to ensure they were applicable. Inclusion criteria included articles which discussed the use of hydrogen peroxide in hip arthroplasties, and all other articles which discussed the use of hydrogen peroxide outside of hip arthroplasties were excluded. In this narrative review, we present the current uses of hydrogen peroxide whilst evaluating its associated risks. We have summarised the key indications and provided a guideline of the use of hydrogen peroxide in hip arthroplasty, as seen in Figure 1.

## Uses of hydrogen peroxide in hip arthroplasties

The use of hydrogen peroxide during hip arthroplasty has always been considered ‘off-label’ by the Medicines and Healthcare products Regulatory Agency (MHRA 2014a). The pursuit of effective debridement techniques within orthopaedic surgeries has led some surgeons to prescribe this treatment with the understanding that they will be held responsible and accountable for their decision (BHS 2015).

As an irrigating solution with effervescence, hydrogen peroxide can mechanically remove tissue debris such as fat, blood and marrow from surface interstices and bony microstructures, which can also increase bony trabeculae porosity (Ackland et al 2009, Yang et al 2016). This debris removal is also important for preventing infections. Since periprosthetic joint infections occur in about 1.7% of primary total hip arthroplasties, reducing infections by using topical adjuvants like hydrogen peroxide can be essential (Ernest et al 2018). With regard to its efficacy, a previous investigation has shown statistically significant reductions in colony forming unit/cm^2^ (CFU/cm^2^) accomplished with several tested chemical adjuvant treatments, including 3% hydrogen peroxide (Ernest et al 2018). It should, however, be noted that this reduction in bacterial colonies may be questionable as the overall concentration of bacteria never fell below 10^5^ CFU/cm^2^ (Ernest et al 2018).

However, hydrogen peroxide treatment has also reduced infections by aiding in the attachment of antibiotics via passivation. Since a surface oxide layer is needed, titanium alloy surfaces are passivated with hydrogen peroxide to attach antibiotics like vancomycin, but this method is corrosive and fails to preserve complex geometries (Ketonis et al 2009). Especially in cases where complex geometries are associated with implant design, passivation by hydrothermal treatment is a better alternative to hydrogen peroxide treatment, as indicated by a comparison between scanning electron microscopy microtopographies of hydrothermally aged titanium alloys and hydrogen peroxide treated titanium alloys (Ketonis et al 2009).

Other purported roles of hydrogen peroxide include the reduction of the most common late complication after cemented joint replacement surgery: aseptic loosening of the prosthesis secondary to cement–bone interface failure (Ackland et al 2009). Maximum tensile pull-out force required to separate the prosthesis from the femoral canal can be used to indicate the presence of aseptic loosening, with a higher force associated with reduced loosening. In one study, significantly higher force was required for pulse-lavage brushing followed by hydrogen peroxide-soaked gauze packing and pulse-lavage brushing alone compared to hydrogen peroxide-soaked gauze packing alone or normal saline irrigation alone, thus indicating that hydrogen peroxide can be used in conjunction with other treatment even if ineffective by itself (Ackland et al 2009). Notably, however, aseptic loosening may be due to a failure at either the prosthesis–cement interface or the cement–bone interface. The former interface however is not affected by hydrogen peroxide use, as by this point in the operation, the hydrogen peroxide solution will have already been used and washed away. Only the cement–bone fixation is strengthened by hydrogen peroxide, aligning with other similar studies, such as an in vitro tensile loading study that achieved better cement fixation when using hydrogen peroxide compared to using normal saline or povidone iodine (Ackland et al 2009, Yang et al 2016). In conjunction with specific techniques, hydrogen peroxide may show synergy with other antiseptics, such as chlorhexidine and dilute povidone-iodine (Flemming et al 2016). Further research into a synergistic approach may additionally allow lower concentrations of hydrogen peroxide to be employed with the same efficacies, in tandem lowering risks of its associated adverse effects.

As a result of induced osteoconductive properties, treatment with hydrogen peroxide has also been found to enhance bone growth, thereby leading to a faster achievement of tight bonding between bones and prostheses than with untreated titanium fiber mesh (Kim et al 2003). However, this proposed role has been undermined by the observation that whilst hydrogen peroxide treatment is statistically superior to normal saline when measuring tensile load, in vivo forces predominantly seem to be compression and shear (Guerin et al 2006). One study has even suggested that hydrogen peroxide actually contributes to aseptic loosening in the long-term; experimentally showing that the fatigue life of polymethyl methacrylate (PMMA) is reduced by a factor of 10, since hydrogen peroxide can affect the material properties of bone cement (Guerin et al 2006). However, no subsequent research supporting this has been published, with various other studies in the literature proposing the opposite (Chung and Jeong 2017).

Furthermore, hydrogen peroxide is thought to have haemostatic properties. This is helpful when lamination at the bone–cement interface, due to bleeding from cancellous bones weakens joint replacement fixations (Yang et al 2016). However regardless, hydrogen peroxide may not be the best option for this role as the reduced mean bleeding by freezing saline is significantly more effective than that of adrenaline solution, saline and hydrogen peroxide (Yang et al 2016).

## Risks

The complications of hydrogen peroxide across surgery are well distinguished; however, reports on the risks of hydrogen peroxide in orthopaedic surgery are relatively sparse (Yang et al 2016). Currently, there is scarce literature with regard to hydrogen peroxide toxicity and air embolism formation in orthopaedic-related literature (Henley et al 2004, Peng et al 2020). A case of cardiac arrest following the use of hydrogen peroxide in a hip arthroplasty has previously been reported (Timperley and Bracey 1989). The underlying cause was thought to be an oxygen embolism due to a combination of the use of hydrogen peroxide in an unvented femoral canal and acrylic bone cement (Timperley and Bracey 1989). A vented cavity involves drilling a hole into the distal cortex of the femur to reduce the intramedullary pressure, thus an unvented cavity would allow the intramedullary pressure to build up intramedullary pressure increasing the risk of fat and air emboli (Dalgorf et al 2003, McHale & Yarlagadda 2014). In an unvented cavity, the increased pressure would lead to rapid absorption of oxygen in the cancellous bone, thus leading to an oxygen embolism (Dalgorf et al 2003, Yang et al 2016). Similar complications have been documented when hydrogen peroxide has been used in other closed cavities in the body (Akuji & Chambers 2017, Zhang et al 2015). However, it should be noted that the volume of oxygen required to cause cardiac arrest is approximately 50ml (Muth & Shank 2000). Considering the volume of oxygen released from hydrogen peroxide is much less, it may be inappropriate to solely assign the cause of the asystole solely to the use of hydrogen peroxide (Lu & Hansen 2017). Yang et al (2016) attributed the other causes of the oxygen embolism to cementing. Regardless, to minimise any risk of such complications, use of hydrogen peroxide in hip arthroplasties should be performed in a vented femoral canal as studies have shown the uncontained cavity liberates the oxygen emboli (Peng et al 2020). Venting did not just mean drilling the distal end of the bone but also included techniques like introduction of a catheter or an aspirator into the cavity. To reduce the risks of an embolism, the hydrogen peroxide gauze needs to be squeezed off and not soaking wet. In addition, avoiding high pressurisation when cementing the femoral canal would help (Lu & Hansen 2017).

It is suggested that hydrogen peroxide has an erosive effect on arthroplasty materials such as titanium alloys and thermal sprayed hydroxyapatite (HA), thus contributing to the destruction of the cement at the cement–bone interface in arthroplasties (Guerin et al 2006). This indicates caution when using hydrogen peroxide in prosthesis made from titanium alloys and HA due to the potential risk of aseptic loosening.

Furthermore, hydrogen peroxide is known to be cytotoxic to various cell types including osteoblasts, chondrocytes, fibroblasts (Röhner et al 2011, Wilson et al 2005).

## Guidelines

Due to the risks associated with its ‘off-label’ use, national guidelines have been variably proposed around the use of hydrogen peroxide in hip arthroplasty. The British Hip Society (BHS) and the Medicines and Healthcare products Regulatory Agency (MHRA) particularly emphasise awareness of associated risks alongside three recommendations (BHS 2015). Firstly, due to the risk of air embolism when employing hydrogen peroxide in closed body cavities or on large/deep wounds (Yang et al 2016), BHS and MHRA recommend use only in vented cavities, employing a suction catheter/aspirator as necessary to achieve this. Secondly, due to reported mortalities correlated to hydrogen peroxide use at high percentage concentrations often ≥3% (Yang et al 2016), the BHS and MRHA recommend use of dilute hydrogen peroxide below 1.5%. It should be noted however that this may compromise its efficacy to an extent, with some evidence suggesting that the catalase enzyme present in gram positive bacteria obfuscates the efficacy of hydrogen peroxide solutions diluted below 3% (McDonnell & Russell 1999). However, taking advantage of the synergy that hydrogen peroxide exhibits with other antiseptic agents may allow retention of efficacy even at a lower concentration around 1.5%, whilst minimising the potential risks of utilising higher concentrations of hydrogen peroxide. Indeed, some have recommended within the literature to follow a hydrogen peroxide soaking period with antiseptics such as 0.3% dilute povidone-iodine or 4% chlorhexidine gluconate, with interspersed saline irrigation steps (Lu & Hansen 2017).

Finally, the use of hydrogen peroxide is only recommended, by Shigematsu et al (2005) on moist (not wet) material, as it could potentiate the chance of embolism formation (Lu & Hansen 2017). Following similar logic, Shigematsu et al (2005) encourage taking sufficient time to wash with saline and dry the region after the use of hydrogen peroxide, prior to insertion of a socket or stem that may increase internal pressure within the relevant cavity and potentiate emboli formation. Specific recommendations based on the interaction between arthroplasty materials and hydrogen peroxide have also been proposed (Shigematsu et al 2005). Due to oxidation of titanium (Ti-6Al-4V) alloys and the formation of grain boundaries by hydrogen peroxide on HA materials that may weaken the fixation power and outcome of surgery, the use of prostheses consisting of these materials with hydrogen peroxide has been cautioned against. To address the same concerns of material erosion, the hydrogen peroxide wound soaking period has also been recommended to be kept minimal, within 1 minute (Shigematsu et al 2005). Finally, under no circumstances should the articular cavity be filled with hydrogen peroxide after reduction as it would not be possible to completely suction the remains in the closed cavity (Shigematsu et al 2005). A summary of the recommendations to use H2o2 can be seen in Figure 1.

The advice from the MRHA to surgeons wanting to use ‘off-licence’ hydrogen peroxide in hip arthroplasties include (MHRA 2014b):

1. Use of hydrogen peroxide would be better for the patient’s needs than any licensed alternative.
2. To create an evidence base using hydrogen peroxide to understand its safety and efficacy.
3. The surgeon will take responsibility for prescribing the hydrogen peroxide and for overseeing the patient’s care, including monitoring and follow-up.
4. The surgeon must record that standard practice is not being followed and the reasons for prescribing ‘off-licence’ hydrogen peroxide.

The MRHA also discusses the best practice for communication to discuss the use of hydrogen peroxide with the patient (MHRA 2014b):

1. The surgeon must provide sufficient information about hydrogen peroxide to enable them to make an informed decision.
2. The surgeon must explain the reasoning behind prescribing hydrogen peroxide.

Following review of the available evidence, we have devised recommendations for hydrogen peroxide in hip arthroplasty:

1. Only use in a vented cavity.
2. Take caution if using with HA/titanium alloy implants.
3. Use dilute concentration (≤1.5%).
4. Soaking period should be ≤1 minute.
5. Apply hydrogen peroxide on moist (not wet) material, for example ribbon gauze.
6. After use, wash (cavity or wound) with saline and dry.
7. Consider hydrogen peroxide use with subsequent antiseptic solutions (eg: povidone iodine/chlorhexidine gluconate).
8. Use before insertion of socket/stem.
9. The patient must be provided with sufficient information in order to allow for an informed decision and must discuss the reasoning behind the use of hydrogen peroxide.
10. The surgeon/doctor must be satisfied that no licenced alternative can meet the patient’s need better than hydrogen peroxide.

## Conclusion

In conclusion, hydrogen peroxide constitutes a cheap, widely available antiseptic agent that offers a variety of potential benefits in its use to prepare trabecular bone for hip arthroplasty surgery. Despite being associated with risks of producing oxygen emboli, hydrogen peroxide is commonly used to mechanically remove debris to prevent infection. In order to minimise the complications associated with hydrogen peroxide, appropriate precaution should aid minimisation of these risks. Due to the variety of benefits offered, hydrogen peroxide use will likely continue. However, it is not advised to use hydrogen peroxide for its haemostatic properties as there is limited evidence to support its efficacy while alternatives with fewer side effects are more effective. Thus, we have aggregated the existing recommendations and evidence to provide guidance for hydrogen peroxide use in hip arthroplasty for the preparation of trabecular bone.

*No competing interests declared*
